# High performance organic light-emitting diodes employing ITO-free and flexible TiO_*x*_/Ag/Al:ZnO electrodes†

**DOI:** 10.1039/d1ra02214h

**Published:** 2021-05-12

**Authors:** Lukas Kinner, Theodoros Dimopoulos, Giovanni Ligorio, Emil J. W. List-Kratochvil, Felix Hermerschmidt

**Affiliations:** Humboldt-Universität zu Berlin, Institut für Physik, Institut für Chemie & IRIS Adlershof Zum Groβen Windkanal 2 12489 Berlin Germany felix.hermerschmidt@hu-berlin.de; AIT Austrian Institute of Technology, Center for Energy, Photovoltaic Systems Giefinggasse 6 1210 Vienna Austria theodoros.dimopoulos@ait.ac.at; Helmholtz-Zentrum Berlin für Materialien und Energie GmbH Hahn-Meitner-Platz 1 14109 Berlin Germany

## Abstract

The broad application of flexible optoelectronic devices is still hampered by the lack of an ITO-free and highly flexible transparent electrode. Dielectric/metal/dielectric (DMD) transparent electrodes are promising candidates to replace ITO, especially in flexible devices due to their mechanical stability to bending, high optical transmittance and low sheet resistance (<6 Ω sq^−1^). This paper reports on organic light emitting diodes (OLEDs) employing a DMD electrode, specifically TiO_*x*_/Ag/Al:ZnO (doped with 2 wt% Al_2_O_3_) fabricated by sputter deposition, together with a solution-processed organic polymeric emitting layer. The electrodes were sputtered without substrate heating on rigid glass and flexible polyethylene terephthalate (PET). The results showed that the OLED devices on the DMD electrodes outperform the OLEDs on commercial ITO substrates in terms of maximum luminance as well as current efficacy. Specifically, DMD-based devices achieve up to 30% higher current efficacy on glass and up to 260% higher efficacy on PET, as compared to the ITO-based reference devices. Maximum luminance reaches up to 100 000 cd m^−2^ for the DMD-based OLEDs on glass and 43 000 cd m^−2^ for those on PET. This performance is due to the low sheet resistance of the electrodes combined with efficient light outcoupling and shows the potential of DMDs to replace ITO in optoelectronic devices. This outstanding type of optoelectronic device paves the way for the future high throughput production of flexible display and photovoltaic devices.

## Abstract 2

A flexible ITO-free structure based on nano-engineered transparent TiO_*x*_/Ag/Al:ZnO electrodes is used for the first time in solution-processed OLEDs.

## Graphical abstract

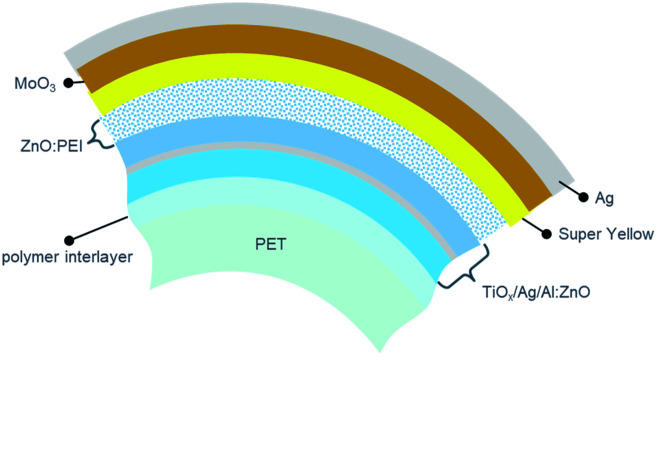

## 1. Introduction

Since organic optoelectronic devices have entered the consumer market, indium tin oxide (ITO) has been the dominant transparent electrode (TE) in the industry.^1^ ITO deposited on glass by sputtering, combines high optical transmittance (>85%), low sheet resistance (<20 Ω sq^−1^) and chemical stability, all necessary requirements for optoelectronic device applications.^2^ On the other hand, the brittleness of ITO and the scarcity of indium are serious disadvantages.

Optoelectronic devices, such as organic light emitting diodes (OLEDs) and organic photovoltaics, can be produced at high throughput by roll-to-roll (R2R) processing on flexible and low-cost substrates, such as polyethylene terephthalate (PET).^3^ However, flexible device processing and operation place additional challenges on the transparent electrodes. The requirement to reduce the deposition temperature and rate to a regime compatible with the flexible substrates, adversely affects the conductivity of the sputtered ITO.^4^ Additionally, ITO prohibits extensive mechanical bending, since it is a brittle material.^5^ In terms of optical losses, the PET/ITO interface shows high internal reflections, due to waveguide trapping of internally emitted light in ITO/organic layers, which decreases light outcoupling efficiency in devices.^6^

To overcome these drawbacks of ITO, many alternative flexible TEs have been developed. Some of the most prominent are metal grids,^7–9^ silver nanowires^10,11^ and carbon-based materials,^12–14^ which are applied by solution-based processing techniques (such as inkjet printing, slot-die coating, blade coating, *etc.*). In addition to these, there are vacuum-based techniques to produce TE alternatives, which offer high mechanical flexibility, low processing temperature and ITO-free composition. In particular, dielectric/metal/dielectric (DMD)^1^ layers can be R2R-sputtered, which offers fast and hence low-cost production.^15^ As metal layers, Ag, Cu or Au are used, with a thickness in the range of 5–15 nm, offering low optical losses, high conductivity and ductility. Of the three metals, Ag provides the best trade-off between optical and electrical properties, as well as cost, and is thus the most widely used. By sandwiching the metal between two dielectrics, optical reflection losses are suppressed and light outcoupling is enhanced.^2^ A wide range of dielectrics have been used in the literature, based on their optical (refractive index) and electronic (work function, bandgap, electron affinity) properties, in order to match specific device architectures. A widely used dielectric is Al-doped ZnO, here referred to as Al:ZnO, and often referred to as AZO in literature.

For TE deposition, sputtering is the state-of-the-art technique used in industry to deposit ITO. This technique offers many advantages, such as control of the layer thickness at the nanometer scale, high deposition rates, high layer adhesion and homogeneity, adjustment of the layer stoichiometry, and a very wide spectrum of processable materials, from metals to insulators.^16^

Gentle *et al.* used a sputtered Al:ZnO/Ag/Al:ZnO TE and a solution-processed emitting layer, whereas as hole injection layer, a 10 nm-thick MoO_3_ layer was evaporated on the TE.^17^ The emitting layer consisted of spin-coated PDY-132 “Super Yellow” (from Merck, Germany). The OLEDs with the DMD (on glass substrate) showed 50% higher external quantum efficiency than reference devices on ITO. The higher efficiency is achieved due to maximized light outcoupling by the electrode architecture.

Further improvement of the sputtered DMD electrode with Ag was achieved by replacing the Al:ZnO layer adjacent to the substrate with TiO_*x*_. The TiO_*x*_/Ag/Al:ZnO electrode achieved highest transmittance and lowest *R*_sh_ on glass^18^ and PET^19^ that was modified through the use of polymer layers. In the latter case, the average transmittance in the 400–700 nm range is the highest reported for a DMD electrode on PET, with 85.1%, whereas in the same wavelength range the DMD electrode on glass featured 88.1% transmittance. On both substrates the sheet resistance was 5.7 Ω sq^−1^.^19^

To the best of the authors' knowledge, such a TiO_*x*_/Ag/Al:ZnO DMD architecture has not been implemented in solution-processed OLEDs before. This paper therefore reports on OLEDs using solution-processed electron injection and light-emitting layers as well as sputtered transparent electrodes. On top of the sputtered Al:ZnO layer, we processed a ZnO nanoparticle (NP) layer that includes polyethylene imine (PEI) to lower the work function. The OLEDs were fabricated on both rigid and flexible substrates employing glass and PET, respectively. The combination of the sputtered TiO_*x*_/Ag/Al:ZnO layers with the ZnO:PEI layer is abbreviated with TAZ for the sake of clarity. All solution processing was carried out in ambient air. It is shown that high performance OLED devices are obtained with the use of the TAZ on both glass and PET, with current efficacies higher than the corresponding commercial ITO-based reference devices.

## 2. Experimental techniques

The OLED devices were fabricated on rigid and flexible carrier substrates, employing glass and PET films. For the devices on rigid substrates, 20 × 15 mm^2^ bare glass substrates (from Ossila, UK) were cleaned in a 2% Hellmanex–ultrapure water, solution in an ultrasonic bath, then rinsed with ultrapure water, sonicated in acetone and isopropanol and then blown dry with nitrogen. For the devices on flexible substrates, PET substrates (Melinex® 504 with one side treated for improved adhesion) were first cut into 20 × 15 mm^2^ pieces and then cleaned in the same way as glass, except for the acetone sonication. Modified PET was obtained by static spin-coating of Amonil® MMS10 (from AMO GmbH, Germany) on the PET substrate at 1000 rpm for 120 s, yielding a polymer thickness of 250 nm (see ref. 19 for more details on the modification of PET). In short, Amonil® makes the rough PET surface glass-like and lowers the RMS roughness of PET significantly, which leads to superior film formation of the sputtered layers. For the sake of clarity, the modified PET will simply be referred to as PET in the remainder of this manuscript.

Metals and dielectrics were sputtered at direct current (DC) magnetron mode in a Leybold Univex 450C cluster tool, with a base pressure in the deposition chamber of 1.4 × 10^−7^ mbar. TiO_*x*_ was deposited by reactive sputtering from a Ti target in Ar/O_2_ (80/20) plasma, at 120 W sputter power and 1.0 × 10^−3^ mbar pressure, yielding a sputter rate of 0.014 nm s^−1^. Ag was sputtered at 40 W, in pure Ar plasma and 1 × 10^−3^ mbar pressure, at a rate of 0.5 nm s^−1^. Finally, Al:ZnO was sputtered in pure Ar from a ZnO target with 2 wt% Al_2_O_3_ at 1 × 10^−3^ mbar and 60 W, resulting in a rate of 0.28 nm s^−1^. All targets were 101.6 mm in diameter and their distance to the substrate was 100 mm. All films were deposited without substrate heating. In the following, the resulting layer thickness is denoted by a subscript, *e.g.* Al:ZnO_51_ stands for Al:ZnO layer with 51 nm thickness. The structuring of the electrodes is described in the ESI (Fig. S1(a–e)).†

The performance of the OLEDs was compared with that of OLEDs based on commercially available ITO. For this purpose, patterned ITO-coated glass (from Psiotec, UK) and patterned ITO-coated PET (from Psiotec, UK) were used as reference. The sheet resistance of the ITO film on glass and PET is 15 Ω sq^−1^ and 60 Ω sq^−1^, respectively. The size and patterning of these substrates was the same as for the glass substrates.

All coated substrates were plasma-treated in a Femto Diener Plasma chamber at 150 W with 0.35 mbar Ar partial pressure for 5 minutes to improve cleanness and wettability of the surface. For the fabrication of the OLED (Fig. S1(f)),† an electron injection layer based on ZnO NPs and PEI was employed.^20^ The electron injection was obtained by employing a 2 : 1 by volume solution of ZnO NPs in IPA (from Genesink, France product number: H-SZ91066) and PEI (Sigma Aldrich product code: 408727).^21^ The films were statically spin-coated at 2500 rpm for 60 seconds and then heated on a hot-plate for 10 minutes at 110 °C. As active layer, the emitting layer PDY-132 “Super Yellow” (Merck, Germany) was employed. The polymer was dissolved at 5 g L^−1^ in toluene and statically spun at 2500 rpm for 60 seconds, without any heating applied. The excess solution was removed from the edges of the sample using a cotton pad immersed in toluene. As top contact, 10 nm MoO_3_ and 200 nm Ag were evaporated through a shadow mask, forming the OLED pixels (marked with dashed rectangles in Fig. S1(f)).†

Bending tests were performed with a MARK 10 ESM 3 tension/compression force setup at a speed of 1 m min^−1^. The bending radius was 4 mm for compressive and for tensile stress. The electrical resistance of the samples was measured from one edge to the opposite edge of the sample, along the strain direction. Silver paste, combined with Cu adhesive tape, was used as contacts. The resistance was measured in the relaxed state at intermittent numbers of bending cycles.

For the optical transmittance measurements, a Bruker Vertex 70 Fourier transform spectrometer, equipped with a visible light source was used. Transmittance spectra were measured at normal light incidence and include the substrate. For the wavelength range 330–550 nm a GaP-detector was used and for the range 550–1150 nm a Si detector. The surface topography was measured by scanning force microscopy (Molecular Imaging, PicoPlus) in tapping mode, using PointProbe® Plus Non-Contact/Tapping Mode–High Resonance Frequency–Reflex Coating (PPP-NCHR) tips from Nanosensors™. The resulting images were processed using the software Gwyddion. The sheet resistance of the samples was measured with a 4-point, in-line probe (Nagy SD – 600). Electroluminescence (EL) spectra were measured with an ocean optics CS2000 spectrometer. IV curves were recorded using a Keithley 2450 source measurement unit, together with a Konica Minolta LS-160 luminance meter for luminance measurements, in a customized setup.

X-ray photoelectron spectroscopy (XPS) was performed in a JEOL JPS-9030 photoelectron spectrometer system, employing monochromatic Al Kα (1486 eV) as excitation source. Ultra-violet photoelectron spectroscopy (UPS) spectra were obtained using the JEOL JPS-9030 employing as exciton energy an H-Lyman-a lamp from Excitech.^22^ The secondary electron cut-off (SECO) was measured with a bias voltage of 10 V. The energy position of the SECO and the binding energy onset of the valence band were determined through linear extrapolation.

**Fig. 1:**
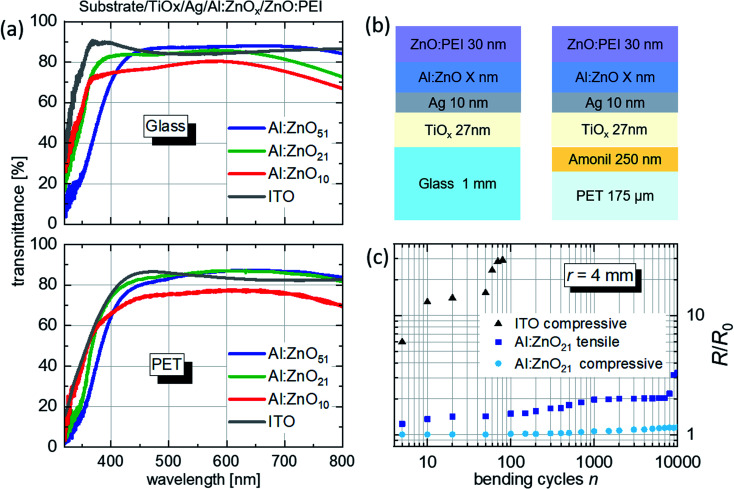
(a) Transmittance spectra of the TAZ and the ITO electrodes including the glass (top graph) and PET (bottom graph) substrates. (b) Schematic architecture of the TAZ electrodes on glass and PET, with varying Al:ZnO thickness (not to scale). (c) Bending test results, showing the superiority of the TAZ electrodes on PET over ITO. The bending radius *r* is 4 mm.

To simulate the fraction of emitted optical power, Lumerical's Device Suite software was used. The fraction of emitted optical power is defined as the power that escapes into the air above the device in relation to the fraction of optical power generated in the active material. A 2-dimensional model of the given OLED architectures was drawn within the software, after which a finite-difference time-domain (FDTD) algorithm was applied, yielding the ratio of outcoupled light power in percent. The layer thicknesses were chosen as in the schematic OLED representations in Fig. 1(b), while the optical parameters such as *n* and *k* were taken from the literature.^19^

## 3. Results and discussion

We have reported in our previous work that for a Ag layer thickness of 10 nm, the TiO_*x*_ and Al:ZnO layer thicknesses that maximize the transmittance, are 27 and 51 nm, respectively.^19^ Given the presence of the additional 30 nm-thick ZnO:PEI layer, in order to optimize the electrode transmittance a systematic investigation was conducted to identify the optimum Al:ZnO layer thickness. For this purpose, three different Al:ZnO thicknesses of 51, 21 and 10 nm were used for the experiments. Fig. 1(a) shows the optical transmittance spectra for the different Al:ZnO thickness of the TAZ electrodes (TAZ includes the ZnO:PEI layer) on glass (top) and on PET (bottom). The figure reports also the spectra collected from the reference ITO electrodes (ITO electrodes also include the ZnO:PEI layer) on glass and PET. The layer sequence of the electrodes is schematically shown in Fig. 1(b). In general, the different electrode architectures yield similar transmittance values, independent of the chosen substrate. Table 1 reports the summary of the physical properties for the electrodes fabricated on glass and PET, compared to the ITO reference devices.

**Table 1:** Optical transmittance and sheet resistance of the TAZ electrodes with varying Al:ZnO thickness and ITO on glass and PET substrates

	Glass					PET				
	*T*_vis_ [%]	*T*_550_ [%]	*R*_sh_ [Ω sq^−1^]	*Φ*_vis_ [10^−3^ Ω^−1^]	*Φ*_550_ [10^−3^ Ω^−1^]	*T*_vis_ [%]	*T*_550_ [%]	*R*_sh_ [Ω sq^−1^]	*Φ*_vis_ [10^−3^ Ω^−1^]	*Φ*_550_ [10^−3^ Ω^−1^]
Al:ZnO_51_	86.2	87.4	5.5	41.2	47.3	83.6	85.8	5.5	30.3	39.3
Al:ZnO_21_	84.2	85.4	5.5	32.5	37.5	84.8	86.2	5.5	35.0	41.2
Al:ZnO_10_	78.2	80.1	5.5	15.5	19.7	75.3	76.6	5.5	10.7	12.7
ITO	85.1	84.0	15.0	13.3	11.6	83.9	84.6	60.0	2.9	3.1

Table 1 reports the summary of the physical properties for the electrodes fabricated on glass and PET, compared to ITO reference electrodes. For the glass substrate, the average transmittance in the 400–700 nm range (*T*_vis_) of the electrodes with Al:ZnO_51_ (blue line) is higher than the transmittance of the electrodes employing ITO (gray line). With the reduction of the Al:ZnO thickness to 21 nm (green line), higher optical losses occur in the visible, while a transmittance increase is observed for short wavelengths, below 450 nm. This reduces *T*_vis_ to slightly below the value obtained for the ITO electrode (Table 1). However, the *T*_550_ value (transmittance at 550 nm) remains higher than the one for ITO. The electrode with Al:ZnO_10_ (red line) has a significantly lower transmittance than the rest of the TAZ electrodes and ITO. At this low Al:ZnO thickness, the layer showed the worst reflection suppression during the transmittance measurement in air, and thereby yielded the lowest transmittance values. Similar observations can be made for the electrodes on the PET substrate, with the difference that the transmittance for Al:ZnO_21_ surpasses the one for Al:ZnO_51_ (Table 1).

Additionally, Table 1 includes Haacke's figure-of-merit *Φ* = (*T*^10^/*R*_sh_),^23^ using either the average transmittance *T* = *T*_vis_ (*Φ*_vis_) or the transmittance at 550 nm *T* = *T*_550_ (*Φ*_550_). According to this parameter, the electrodes generally show increased performance with thicker Al:ZnO layers. Finally, regarding sheet resistance, all electrodes on glass employing the TAZ trilayer show *R*_sh_ < 6 Ω sq^−1^, while the ITO TE has *R*_sh_ = 15 Ω sq^−1^ (Table 1). The sheet resistance is therefore independent of the Al:ZnO thickness, determined by the 10 nm-thick Ag layer. The TAZ electrodes on PET show the same sheet resistance as on glass, while the ITO electrode on PET has *R*_sh_ = 60 Ω sq^−1^ (Table 1).

To fully implement the electrodes on flexible substrates it is necessary to investigate the mechanical stability of the electrode during bending. Bending tests were therefore performed on the DMD layers on PET at a bending radius of *r* = 4 mm. With a PET substrate thickness of *d* = 175 μm, this bending radius corresponds to a 2.2% tensile/compressive film strain (*ε*) according to the formula *ε* = *d*/2*r*.^24^ In Fig. 1(c) the results of the bending tests are shown. *R* is the resistance after intermittent numbers of bending cycles and *R*_0_ is the initial resistance of the TE, respectively. After >10 000 bending cycles of compressive bending, almost no change in the resistance is observed for the TAZ electrode. For tensile bending, a 3-fold increase is observed after 10 000 cycles. The higher change of *R*_sh_ in tensile bending compared to compressive is to be expected since tensile bending is more strenuous for the film.^25^ For the ITO electrode reference on PET, a 30-fold resistance increase is observed after 70 compressive bending cycles. The high flexibility of the TAZ electrode is caused by the ductility of the 10 nm thick Ag layer.^24^

**Fig. 2:**
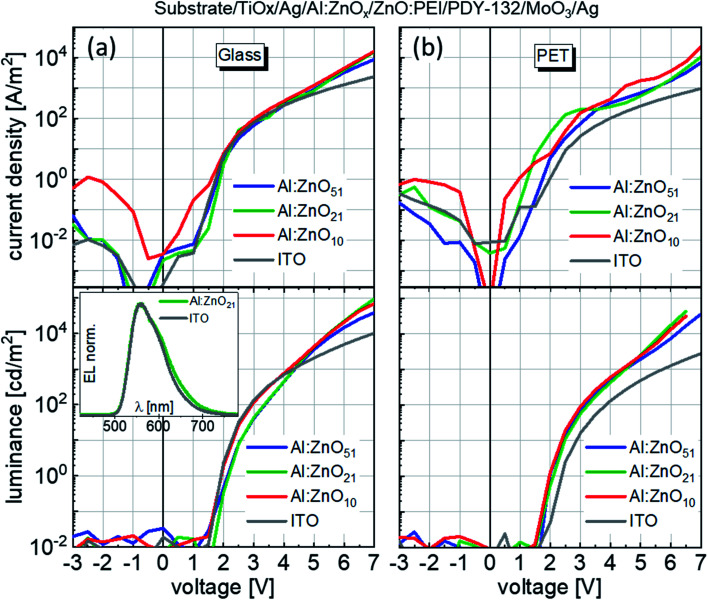
Current–voltage and luminance–voltage characteristics of OLEDs on (a) glass and (b) PET substrates. The inset in (a) shows electroluminescence spectra for two OLEDs, one on ITO and the other on the Al:ZnO_21_ electrode.

Having shown the promising electrical, optical and mechanical parameters of the TAZ electrodes, we proceeded into their implementation into OLED devices. Fig. 2 shows the current density–voltage (*J*–*V*) and luminance–voltage (*L*–*V*) characteristics of the devices employing the TAZ electrodes and ITO, on glass (Fig. 2(a)) and PET (Fig. 2(b)). On glass, the TAZ devices with Al:ZnO_51_ and Al:ZnO_21_ show similar electrical characteristics as the ITO reference device, in the window from −3 to 4.5 V. For *V* > 4.5 V, TAZ devices show higher currents than the ITO device, which is due to their significantly lower *R*_sh_. The luminance output of the devices follows closely the trend of the *J*–*V* curves: below 4.5 V the luminance of the different devices is similar and above 4.5 V TAZ devices show superior performance. The differences in performance, appearing at high bias, are due to the differences in electrode sheet resistance (Table 1). Luminance maximum is achieved for the device on glass with Al:ZnO_21_, with almost 100 000 cd m^−2^. The highest luminance for devices with Al:ZnO_51_ is 76 000 cd m^−2^ and for Al:ZnO_10_ 70 000 cd m^−2^. Devices on ITO achieve a maximum luminance of 49 000 cd m^−2^, *i.e.* considerably lower than any TAZ device. The 3 times higher *R*_sh_ value of the ITO-based devices leads to a faster joule heating, which causes thermal failure of the emitting layer at lower luminance values, compared to the TAZ based devices.^20^

TAZ-based devices on PET (Fig. 2(b)) show higher currents in comparison to ITO-based devices for the entire voltage regime, which is translated to a higher luminance. The difference in current density and luminance between TAZ- and ITO-based devices increases with the voltage, due to the increasing influence of the electrode sheet resistance, which is a factor 12 higher for the ITO than for the TAZ. The device with Al:ZnO_51_ shows the same luminance values as the device with Al:ZnO_21_, while the device with Al:ZnO_10_ shows the lowest luminance. Still all TAZ device samples display a higher luminance than those incorporating ITO. The electrical and luminance characteristics of the devices on PET are similar to devices on glass, except for the absolute luminance values, which are lower on PET (Table 2). We attribute this difference in luminance between glass and PET to the difference in electrode performance, as shown by Haacke's figure of merit above. Independent of the substrate and Al:ZnO thickness, however, all devices showed similar electroluminescence spectra. Such spectra are shown in the inset of Fig. 2(a) for an ITO and Al:ZnO_21_ device on glass.

**Table 2:** Performance figures of OLEDs based on the TAZ electrodes with varying Al:ZnO thickness and ITO, on glass and PET substrates

	Glass					PET				
	*V*_T_ [V]	*L*_max_ [cd m^−2^]	*L*_6V_ [cd m^−2^]	*n*_max_ [cd A^−1^]	*n*_10000_ [cd m^−2^]	*V*_T_ [V]	*L*_max_ [cd m^−2^]	*L*_6V_ [cd m^−2^]	*n*_max_ [cd A^−1^]	*n*_10000_ [cd m^−2^]
Al:ZnO_51_	1.7	75 729	8000	5.23	4.52	2.0	43 410	7300	5.48	4.54
Al:ZnO_21_	1.9	99 910	23 000	6.27	5.74	2.1	42 629	17 400	9.78	7.60
Al:ZnO_10_	1.6	70 294	21 000	5.14	4.36	1.9	31 025	13 100	4.22	3.37
ITO	1.7	49 314	5000	4.67	4.36	2.4	17 650	1300	3.75	3.64

Table 2 summarizes key performance figures for the devices on glass and PET, namely the turn-on voltage at 1 cd m^−2^ (*V*_T_), maximum luminance (*L*_max_), luminance at 6 V (*L*_6V_), maximum efficacy (*n*_max_) and the efficacy at 10 000 cd m^−2^ (*n*_10000_). According to Table 2, the devices with Al:ZnO_21_ show the highest performance values, which leads to the highest efficacy values for this device and will be discussed in detail.

**Fig. 3:**
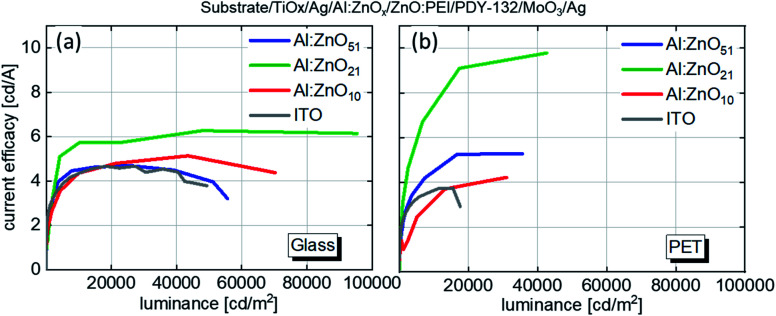
Current efficacy of OLEDs based on TAZ electrodes with varying Al:ZnO thickness and ITO on glass (a) and PET (b) substrates.

In Fig. 3(a) and (b) the current efficacies are plotted *versus* the luminance values of the OLEDs with TAZ electrodes, as well as with ITO electrodes on glass and PET, respectively. On glass, the TAZ electrode devices show behavior that depends on the Al:ZnO thickness. Outstanding performance is achieved for the device with Al:ZnO_21_. This device features a current efficacy at 10 000 cd m^−2^ of 5.74 cd A^−1^, while the ITO reference device has only 4.36 cd A^−1^. In contrast to the ITO device, the Al:ZnO_21_ device shows stable efficacy values, up to almost 100 000 cd m^−2^, with a maximum efficacy of 6.27 cd A^−1^ at 50 000 cd m^−2^. At 100 000 cd m^−2^ it shows 6.14 cd A^−1^–only slightly lower, although twice as bright. The device with Al:ZnO_10_ shows a maximum efficacy of 5.48 cd A^−1^, also higher than the ITO reference, whereas the efficacy of the Al:ZnO_51_ device follows closely the one of ITO. Similar current efficacies (6.1 cd A^−1^) were reached in the literature, but at far lower luminescence values (1000 cd m^−2^).^26^

On PET, the best performing device with Al:ZnO_21_ also shows the highest current efficacy, which equals 9.78 cd A^−1^ at 10 000 cd m^−2^. The devices with Al:ZnO_51_ show on PET a slightly higher current efficacy of 7.60 cd A^−1^ compared to glass (5.74 cd A^−1^). Al:ZnO_10_ shows a maximum current efficacy of 4.22 cd A^−1^, which is below its counterpart on glass. Both Al:ZnO_51_ and Al:ZnO_21_ devices show higher efficacies than ITO, which has a maximum efficacy of 3.75 cd A^−1^. Similar devices from the literature fabricated in air on PET/ITO showed current efficacies of up to 6.6 cd A^−1^.^27^ The low *R*_sh_ of the TAZ electrodes employed in the devices leads to high current density, which combined with efficient light outcoupling leads to the observed efficacy and luminance parameters exceeding the performance of the ITO based devices.

To investigate the high efficacy of the Al:ZnO_21_ devices in comparison to the Al:ZnO_10_ and Al:ZnO_51_ devices, optical simulations were conducted, with all 8 different OLED architectures. The simulation yields the ratio between generated light power and outcoupled light power (light outside the device after passing through the layers). The simulation confirmed that devices incorporating Al:ZnO_21_ have the highest power outcoupling efficiency (Table 3).

**Table 3:** Simulated fraction of emitted light power (outcoupled) at 550 nm in relation to generated light power in the active layer

	Glass [%]	PET [%]
Al:ZnO_51_	35.7	19.4
Al:ZnO_21_	48.8	47.3
Al:ZnO_10_	46.7	44.4
ITO	40.3	33.1

Simulated devices on Al:ZnO_10_ show a similar simulated power outcoupling efficiency, but do not meet these values in the experiment, as observed in the electrode performance in Table 1 and device performances in Table 2. This difference in simulated and experimental results for the Al:ZnO_10_ electrode was explored further.

It was anticipated that the morphology of the Al:ZnO_10_ layers may differ from the morphology of the Al:ZnO_21_ layers. To investigate this, SEM images of the electrodes with the different Al:ZnO thicknesses on glass and PET were taken. The SEM images, however, show no significant difference in morphology (Fig. S2†). In addition to the morphological studies, ultraviolet photoemission spectroscopy (UPS) and X-ray photoemission spectroscopy (XPS) data of the electrodes with different Al:ZnO thicknesses (10, 21, 51 nm) on glass and PET were acquired.

UPS was employed to evaluate the electronic structures of the samples. As shown in Fig. S3 and summarized in Table S1,† all the samples display a comparable valence band onset value (3.2 eV ± 0.1 eV). The work function was measured by evaluating the secondary electron cut off (SECO). The samples measured on PET show the same value of 4.3 eV ± 0.1 eV independently of the thickness of the Al:ZnO. The samples deposited on glass display slightly higher (0.1–0.2 eV) values. The different morphology of Al:ZnO grown on PET compared to glass might suggest a change of superficial dipole at the interface. UPS data were acquired for the other layers utilized in the OLED of this work (Fig. S4†) and the results are summarized in the energy level diagram, shown in Fig. S5†.

X-ray photoemission spectroscopy was employed to chemically analyze the top Al:ZnO layer deposited on PET and glass substrates. The normalized signals of O1s and Zn2p core levels are perfectly overlaying, indicating the same chemical configuration among all the samples (see Fig. S6†). This is confirmed by the calculated O/Zn ratio. The values for the three thicknesses and the two substrates are reported in the Table S2.† The Al2p core level was also measured, but no peak was detectable, indicating that the Al amount is below the detection limit for the instrument. All the samples display a low–yet detectable C1s signal; the peak height is however small compared to the noise intensity. The two components can be attributed to C–C and to C–O contribution, hence indicating the fingerprint of the PET, which might slightly contaminate the deposition chamber upon sputtering.

Interestingly, among all the samples only the 10 nm Al:ZnO on glass displays a measurable signal rising from Ag3d. The amount is negligible compared to O or Zn as it is in the order of 0.1% compared to these elements. This might suggest that some portion of the Al:ZnO_10_ contains pinholes allowing the detection of the layer of Ag beneath the film of Al:ZnO_10_ and hampering the otherwise good simulated performance of the Al:ZnO_10_ devices. Therefore, it can be concluded that the devices with the Al:ZnO_21_ on glass and PET show the highest efficacy values, mostly due to an optimum light outcoupling, combined with a pinhole-free layer morphology.

## 4. Conclusion

High performance sputtered TiO_*x*_/Ag/Al:ZnO DMD electrodes on glass and PET substrates have been tested in inverted OLED devices, combined with a ZnO:PEI electron injection layer, a MoO_3_ hole injection layer and “Super Yellow” as light emitting polymer. The superior properties of the TAZ electrodes in terms of OLED performance can be explained for two reasons. First, the simulated light outcoupling and the maximum luminance values of the TAZ electrodes are higher on glass and PET compared to ITO. Second, sheet resistance of the TAZ electrode on glass is a factor of 3 and on PET a factor of 12 lower as compared to ITO on glass and PET, which yields higher currents with lower leakage and less joule heating. On glass, up to 34% higher current efficacy for the devices with TAZ electrode was achieved (compared to ITO devices), while on PET the efficacy was up to 260% higher. The best performance devices implemented 27 nm of TiO_*x*_, 10 nm of Ag, 21 nm of Al:ZnO and 30 nm of ZnO:PEI. Furthermore, the TAZ electrodes provide a dramatic improvement in terms of mechanical stability, being stable for several thousands of compressive and tensile bending cycles. DMD-based electrodes can therefore be targeted to replace ITO in flexible optoelectronic devices.

## Conflicts of interest

The authors declare no competing financial interest.

## Supplementary Material

RA-011-D1RA02214H-s001
