# Supplementary material for: High performance organic light-emitting diodes employing ITO-free and flexible TiOx/Ag/Al:ZnO electrodes
Source: RSC Adv. 2021 May 12;11(28):17324–31. doi: 10.1039/d1ra02214h (PMC9033011; doi:10.1039/d1ra02214h)
Supplement: RA-011-D1RA02214H-s001 [file RA-011-D1RA02214H-s001.pdf]

Electronic Supplementary Material (ESI) for RSC Advances.
This journal is © The Royal Society of Chemistry 2021

ELECTRONIC SUPPLEMENTARY INFORMATION

High performance organic light-emitting diodes employing ITO-free and flexible TiOx/Ag/Al:ZnO electrodes

Lukas Kinner,[a,b] Theodoros Dimopoulos,*[b] Giovanni Ligorio,[a] Emil J. W. List-Kratochvil [a,c] and Felix Hermerschmidt *[a]

[a] Humboldt-Universität zu Berlin, Institut für Physik, Institut für Chemie, IRIS Adlershof, Brook-Taylor-Straße 6, 12489 Berlin, Germany. Email: felix.hermerschmidt@hu-berlin.de

[b] AIT Austrian Institute of Technology, Center for Energy, Photovoltaic Systems, Giefinggasse 6, 1210 Vienna, Austria.

[c] Helmholtz-Zentrum Berlin für Materialien und Energie GmbH, Hahn-Meitner-Platz 1, 14109 Berlin, Germany.

Device fabrication

The in-house sputtered transparent electrodes (TEs, Figure S1 (a)) were patterned by covering a 5 mm-wide strip in the middle of the sample (Figure S1 (b)) with polyimide tape (Kapton®) and using a cotton swab, immersed in 2 % HCl solution, to remove the TE from the non-covered part of the sample (Figure S1 (c)). The samples were rinsed with ultra-pure water and blown dry, before removing the polyimide strip (Figure S1 (d)). Subsequently, 10 nm Cr and 50 nm Au were thermally evaporated through a shadow mask to form 2 mm-wide contact fingers, with 4 fingers on each side of the central TE strip (Figure S1 (e)). The samples were then rinsed with ethanol and iso-2-propanol.

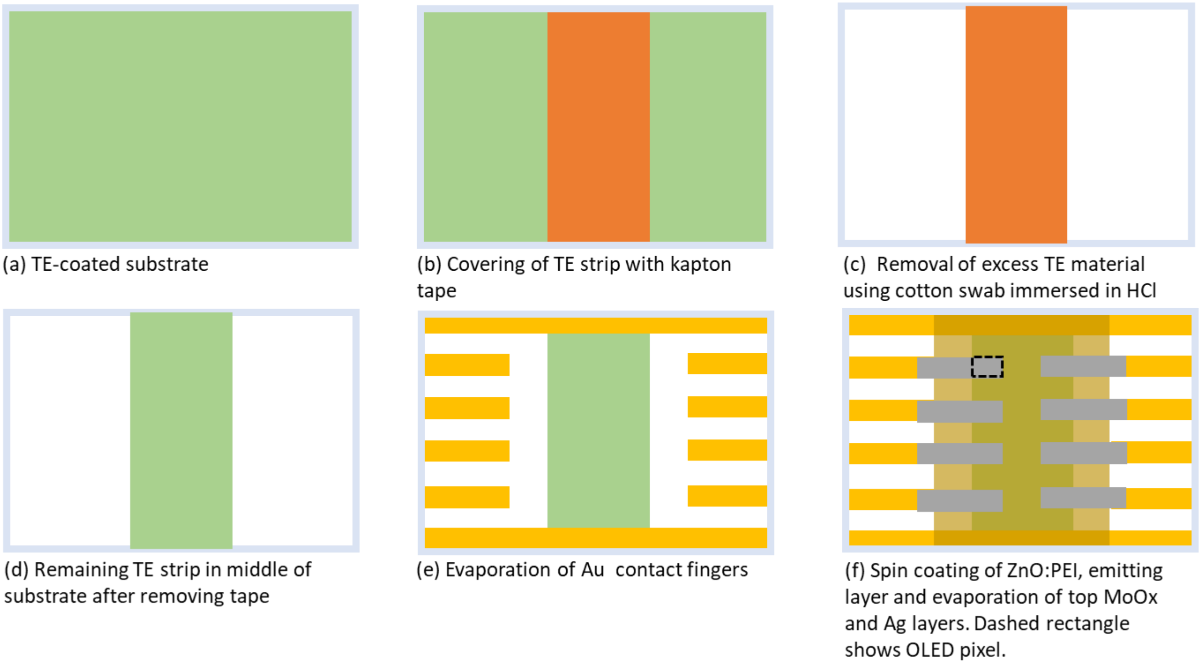

Figure S1. Steps for the TE patterning and fabrication of the OLED devices.

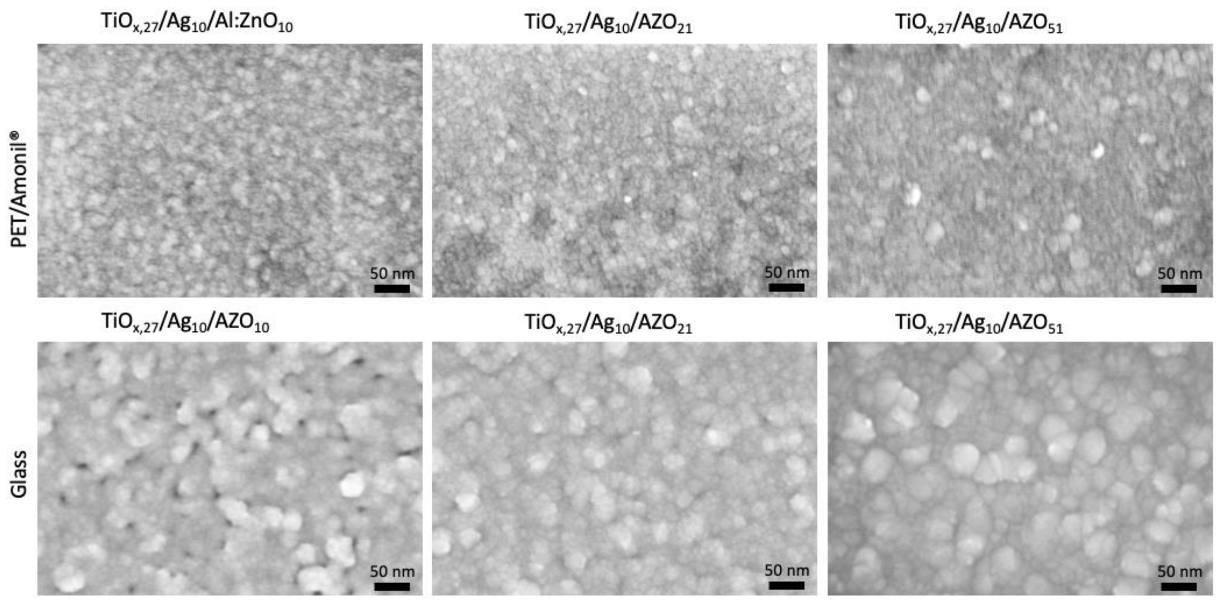

Figure S2. Surfaces of studied electrodes on PET and glass with different Al:ZnO thicknesses, showing a closed morphology of the Al:ZnO layer independent of Al:ZnO thickness.

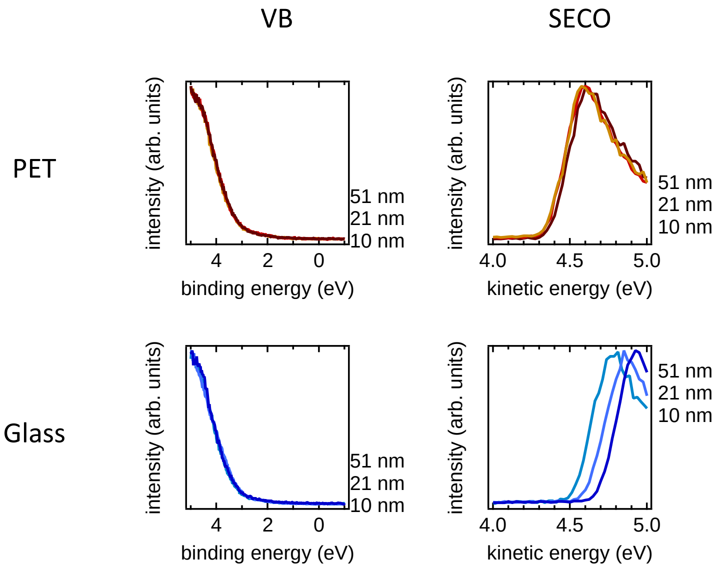

Figure S3. UPS measurements of DMD electrodes on glass and PET with different Al:ZnO thicknesses.

Table S1. The valence band (VB) onset are similar for both substrate types and all thicknesses, while the secondary electron cutoff (SECO) shows only little variation.

	PET		Glass	
	**VB (eV)**	**SECO (eV)**	**VB (eV)**	**SECO (eV)**
10 nm	3.3	4.3	3.3	4.5
21 nm	3.2	4.3	3.2	4.6
51 nm	3.2	4.4	3.3	4.6

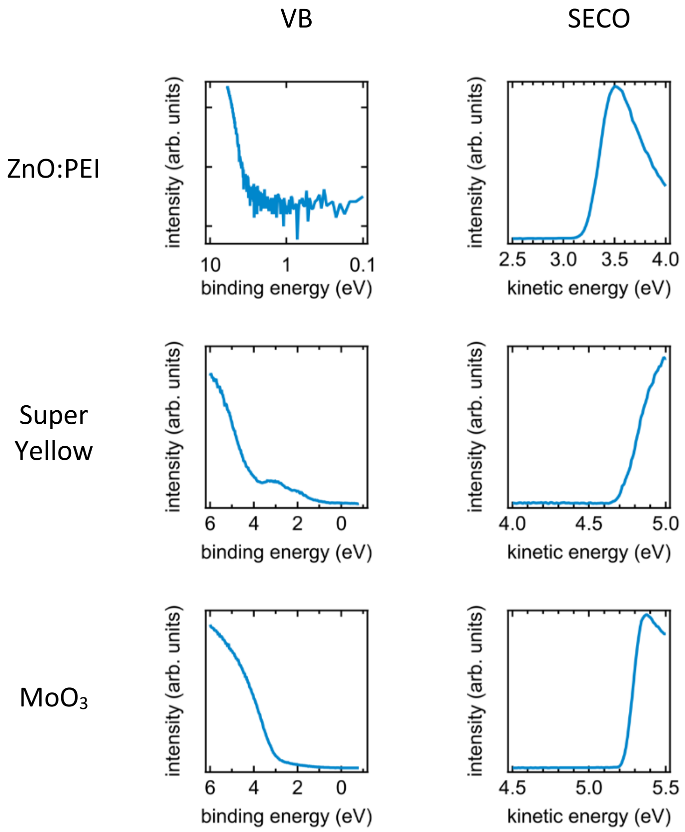

Figure S4. UPS measurements of the layers used in the OLED of this work. The valence band (VB) of the ZnO:PEI layer is shown in a logarithmic form to better visualize the onset.

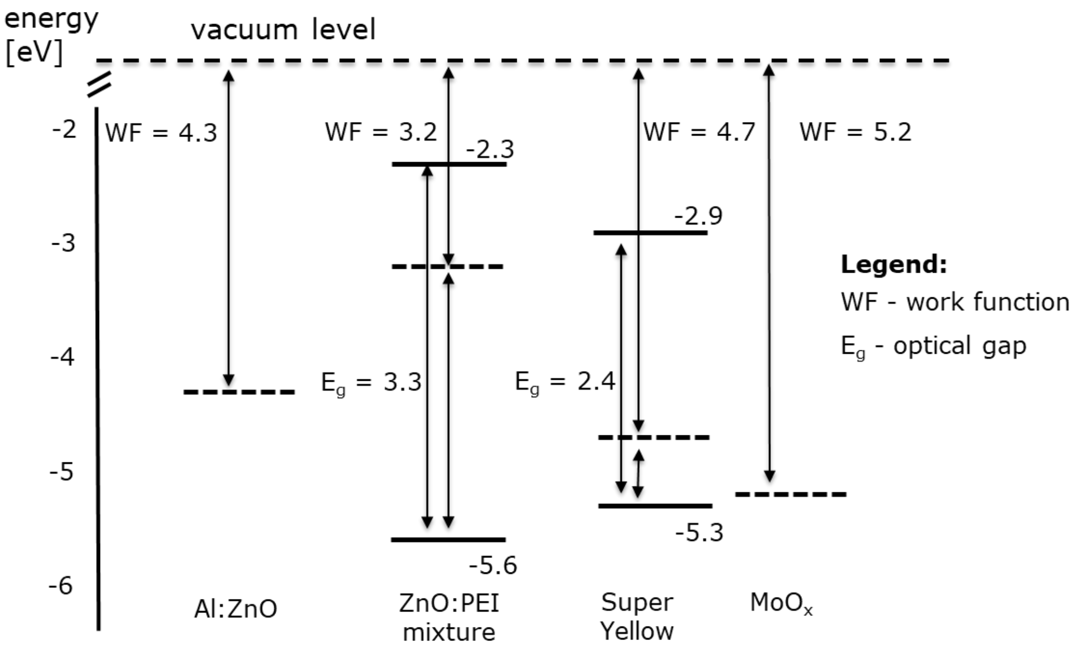

Figure S5: Thin film energy level diagram of studied devices, with work function (WF) determined by UPS (see also Figures S4 and S5) and optical gap (Eg) determined by UV-vis absorption (data not shown). No Fermi level alignment was considered, and all energy levels were considered with vacuum level alignment.

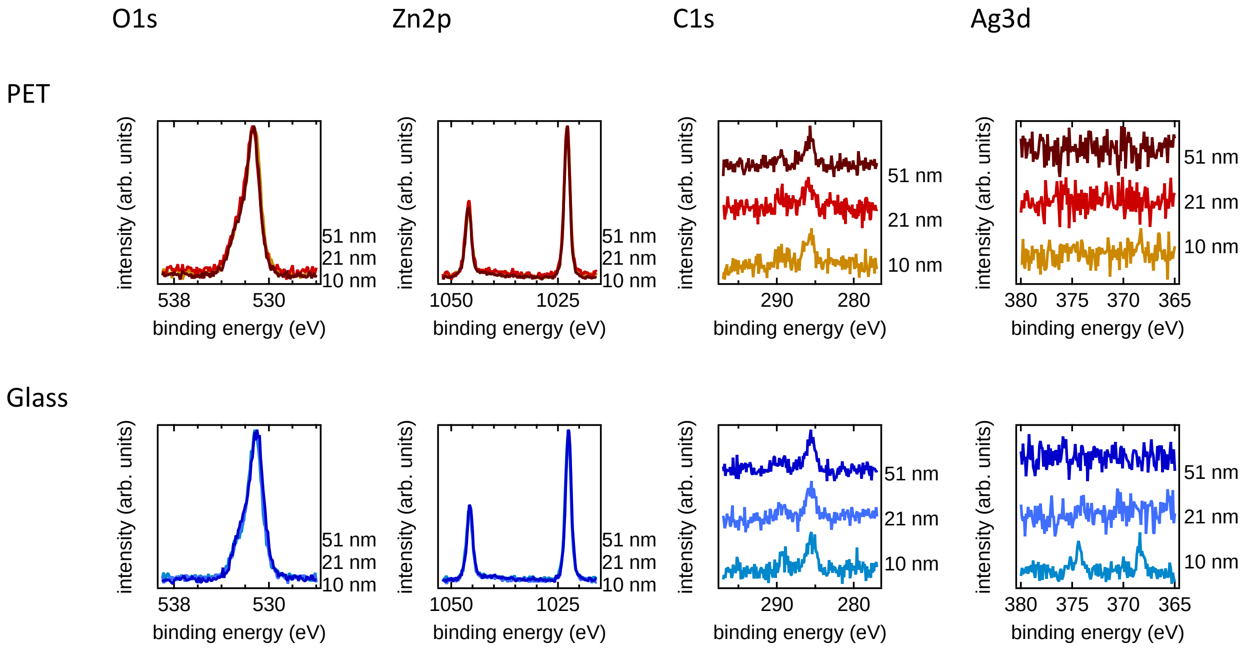

Figure S6. The XPS data reveals no significant differences between PET and glass based layers, with the exception of the 10 nm thick Al:ZnO layer on glass showing two peaks in the Ag 3d signal, indicating possible pinholes in the film.

Table S2. Similar oxygen and Zn ratios are seen across the three substrate thicknesses and two substrate types.

	PET		Glass	
	**O (%)**	**Zn (%)**	**O (%)**	**Zn (%)**
10 nm	65.1	24.9	65.6	34.4
21 nm	64.1	35.9	65.0	35.0
51 nm	63.9	36.1	66.7	33.3
